# Strongyloides hyperinfection in an HIV-positive kidney transplant recipient: a case report

**DOI:** 10.1186/s12879-020-05333-8

**Published:** 2020-08-18

**Authors:** Christina Lai, Matthew Anderson, Rebecca Davis, Lyndal Anderson, Kate Wyburn, Steve Chadban, David Gracey

**Affiliations:** 1 Department of Renal Medicine, RPA Transplantation Services, Royal Prince Alfred Hospital, Missenden Rd, Camperdown, New South Wales 2050 Australia; 2 Kidney Node, Charles Perkins Centre, University of Sydney, Camperdown, 2006 New South Wales Australia; 3 Department of Microbiology and Infectious Diseases, Royal Prince Alfred Hospital, Camperdown, 2050 New South Wales Australia; 4 Central Clinical School, Faculty of Medicine, University of Sydney, Camperdown, 2006 New South Wales Australia; 5 Department of Tissue Pathology and Diagnostic Oncology, Royal Prince Alfred Hospital, Camperdown, 2050 New South Wales Australia

**Keywords:** Strongyloides, Strongyloides hyperinfection, Kidney transplant, Human immunodeficiency virus, HIV, Case report

## Abstract

**Background:**

Strongyloidiasis is caused by the helminth *Strongyloides stercoralis* and is well-recognised amongst transplant recipients. Serious complications, including Strongyloides hyperinfection which is a syndrome of accelerated autoinfection, or disseminated disease, can occur post-transplantation, resulting in significant morbidity and mortality. Here we present the first published case we are aware of, describing post-transplant Strongyloides hyperinfection in an HIV-positive kidney transplant patient. We discuss the diagnostic challenges and the role of pre-transplant screening.

**Case presentation:**

A 58-year-old African-American male, originally from the Caribbean, received a deceased donor kidney transplant for presumed focal segmental glomerulosclerosis. He was known to be HIV-positive, with a stable CD4 count, and an undetectable viral load. Five months post-transplant, he developed gastrointestinal symptoms and weight loss. He had a normal eosinophil count (0.1–0.2 × 109/L), negative serum cytomegalovirus DNA, and negative blood and stool cultures. His Strongyloides serology remained negative throughout. A diagnosis of Strongyloides hyperinfection was made by the histological examination of his duodenum and lung, which identified the parasites. He completed his course of treatment with Ivermectin but exhibited profound deconditioning and required a period of total parenteral nutrition. He was subsequently discharged after a prolonged hospital admission of 54 days.

**Conclusions:**

This case highlights the challenges in diagnosing Strongyloides infection and the need to maintain a high index of clinical suspicion. Non-invasive techniques for the diagnosis of Strongyloides may be insufficient. Routine pre-transplant serological strongyloidiasis screening is now performed at our centre.

## Background

Strongyloidiasis is an infection with global distribution and is associated with high morbidity and mortality amongst transplant recipients, and in immunosuppressed patients [1]. Early treatment of strongyloidiasis is simple and crucial to prevent complications. Strongyloides may be difficult to diagnose; many people carry the dormant parasite without symptoms and non-invasive tests may remain negative, even in the presence of infection. Here we report the first published case of Strongyloides hyperinfection we are aware of in an HIV-positive kidney transplant recipient. We discuss the challenges in confirming the diagnosis, and the role of screening pre-transplant.

## Case presentation

Our case was a 58-year-old African-American HIV-positive male originating from the Caribbean, who received a deceased donor renal transplant for presumed focal segmental glomerulosclerosis, with a background of histologically-proven minimal change disease. He was diagnosed with HIV in 1993 and had a stable, normal, CD4 count (> 400 × 10^6^/L) and an undetectable HIV-viral load. His antiretrovirals (ARVs) included lamivudine (3TC), didanosine (ddI) and lopinavir/ritonavir (LPV/r). During the pre-transplant workup, he was diagnosed with latent tuberculosis (TB), and received 6 months of isoniazid. His Hepatitis B and C, and Stronglyoides serology, performed 4 years prior to transplant, were negative. At the time of transplant, he received standard induction immunosuppression with basiliximab and methylprednisolone, and maintenance immunosuppression of prednisolone, mycophenolate mofetil and tacrolimus. His post-transplant course was complicated by delayed graft function in the context of severe tacrolimus toxicity, as a result of a drug-drug interaction with LPV/r. His ARVs were subsequently modified to raltegravir, 3TC and ddI and his graft function improved. His CD4 count remained stable throughout, with an undetectable viral load.

Five months post-transplant, he presented with a two-week history of abdominal pain, anorexia, diarrhea and weight loss. He had an acute kidney injury with a rise in creatinine from baseline of 167 μmol/L (eGFR 38mls/min/1.73m^2^) to 228 μmol/L (eGFR 26mls/min/1.73m^2^), which resolved with intravenous fluid resuscitation. Initial investigations were non-diagnostic, with a normal eosinophil count (0.1–0.2 × 109/L), negative serum Cytomegalovirus DNA, and negative blood and stool cultures. Faecal PCR tests for Shigella, Salmonella, Campylobacter, and *Clostridium difficile* and faecal culture for other bacterial pathogens were all negative, as were three samples for faecal microscopy for larvae and faecal helminth culture. Serum Strongyloides antibody testing was also negative, with an enzyme-linked immunosorbent assay ratio of 0.74 (ratio < 0.8 negative). HTLV-1/2 serology was negative.

**Fig. 1:**
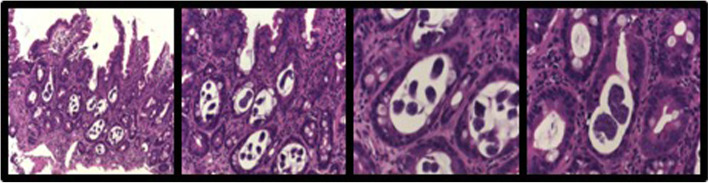
(from left to right): Haematoxylin and Eosin stained sections **a**)× 10 magnification; **b**)× 20; c)× 40; d)× 40. Duodenal biopsy - There was active chronic inflammation and architectural distortion associated with numerous round worm parasites and larvae within crypts

The patient continued to have watery diarrhea. A gastroscopy was performed demonstrating erosive duodenitis with active chronic inflammation. As shown in Fig. 1, there were frequent parasites and larvae within duodenal crypts and at the mucosal surface. The morphology of the parasites confirmed the diagnosis of strongyloidiasis; the patient was commenced on ivermectin 200 mg orally.

**Fig. 2:**
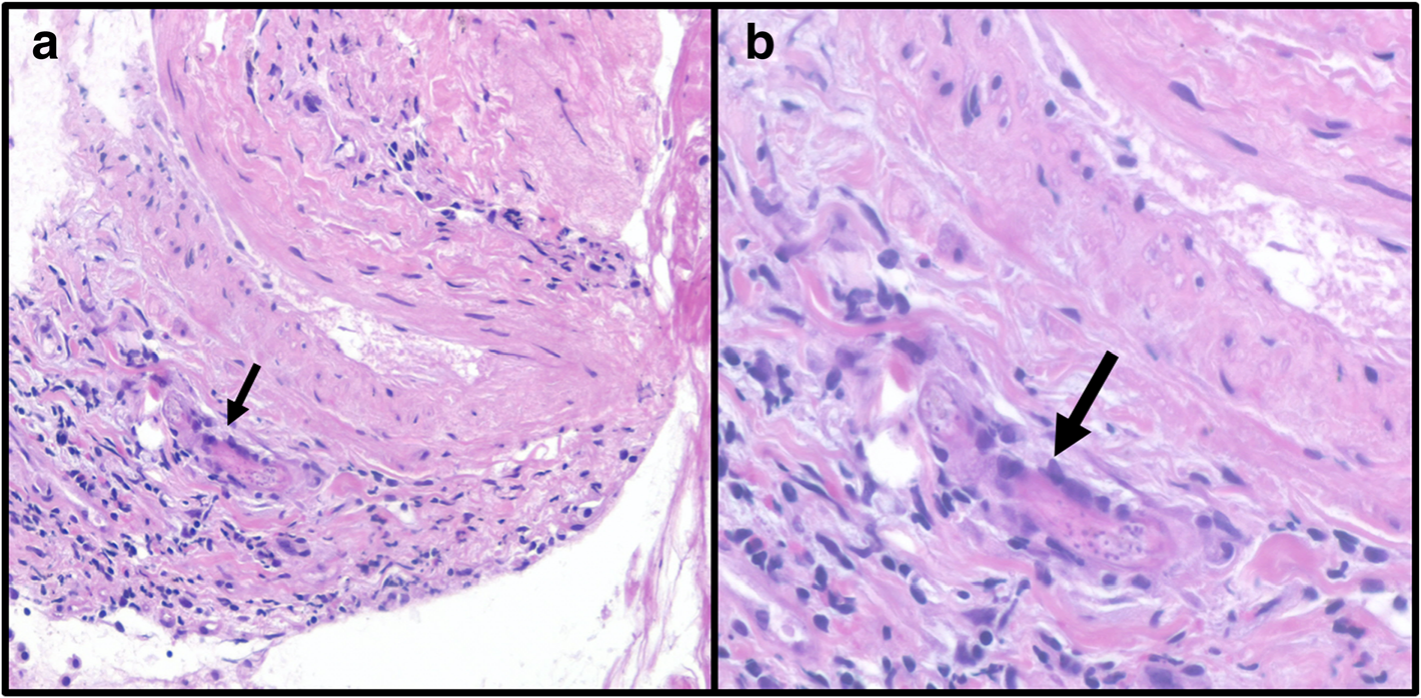
**a** Haematoxylin and Eosin stained lung tissue section showed minimal inflammation and haemosiderin-laden macrophages within alveolar spaces. **b** A single organism (arrow) identified consistent with strongyloidiasis

Despite anti-helminthic therapy, he developed increasing dyspnoea. A computerised tomography (CT) of his chest demonstrated infiltration of the right lower lobe. A bronchoscopy and bronchoalveolar lavage were non-diagnostic. Subsequently, a CT-guided biopsy of the affected area demonstrated inflammatory cells and a single helminth, consistent with Strongyloides hyperinfection, as shown in Fig. 2.

He continued ivermectin for Strongyloides hyperinfection. He exhibited deconditioning and required a period of total parenteral nutrition. Several weeks after completing treatment with ivermectin our patient began to slowly improve, with the resolution of the abdominal pain and diarrhoea. His repeat chest x-ray showed no consolidation. His oral intake increased, and he was eventually able to satisfactorily maintain bodyweight without supplemental feeding. He was subsequently discharged after a prolonged hospital admission of 54 days.

## Discussion and conclusion

Strongyloidiasis is a helminthic disease caused by the nematode parasite *Strongyloides stercoralis*. This has been well-recognised amongst transplant recipients [2–4]. Transplant recipients may suffer serious complications as a result, including Strongyloides hyperinfection syndrome and disseminated disease, with a mortality rate up to 87% if left untreated [5]. The life-threatening complications are often due to a delayed diagnosis. Here we presented a case highlighting the diagnostic challenges in an immunosuppressed patient with negative non-invasive investigations, including negative Strongyloides serology. Our patient is the first case of Strongyloides hyperinfection reported that we are aware of in an HIV-positive kidney transplant patient.

Eosinophilia may be a useful marker of parasitic infection. It is more common in early, acute, Strongyloides infections when worm burden and larval counts are the highest [6, 7]. The degree of eosinophilia has been shown to be predictive of shock and subsequent mortality in immunocompromised patients with Strongyloides hyperinfection [8]. However, eosinophilia may also be less reliable in immunosuppressed transplant patients [1], as was evident in our patient, who maintained a normal eosinophil count throughout.

The current gold standard diagnostic non-invasive test for Strongyloides is serial microscopic examination for larvae in faecal samples [9]. The yield of a single stool specimen for ova and parasites is 15–30%, due to low parasite load and intermittent excretion. The sensitivity may be increased by increasing the number of stool examinations up to seven [10]. These limitations make faecal microscopy a rather suboptimal gold standard. Other techniques reported to have higher sensitivities include agar plate culture, or the Baermann technique [11].

Patients with strongyloidiasis secrete different isotypes of immunoglobulins to combat the parasite. Various serological techniques have been developed to improve the sensitivity in diagnosing strongyloidiasis. These include indirect immunofluorescence microscopy, gelatin particle agglutination, immunoblot and ELISA (enzyme-linked immunosorbent assay). ELISAs are the most widely used tests and have a reported sensitivity of 84–95% and a specificity of 82–100% [5]. False-negative results, however, may occur particularly in immunocompromised patients, as illustrated in this case.

A more recent test may identify *Strongyloides stercoralis* nucleic acids, from either stool or urine samples, usually using polymerase chain reaction (PCR) [9, 12]. Molecular detection of *Strongyloides stercoralis* has improved sensitivity, as compared to serological methods. Even this test may fail however to diagnose those with low larval output.

The diagnosis of strongyloidiasis in our patient was made on tissue histology. In patients with gastrointestinal symptoms, the morphologic changes of Strongyloides colitis may mimic idiopathic inflammatory bowel disease, resulting in diagnostic error [13]. Distinctive features of Strongyloides colitis include the presence of skip lesions, involvement of the submucosa, disease attenuation toward the distal colon, eosinophil-rich inflammation with eosinophilic micro-abscess formation and extra-crypt micro-abscess [13]. These findings should prompt a careful search for larvae to definitively make the diagnosis.

The limitations of non-invasive diagnostic tests make it challenging to diagnose strongyloidiasis in a timely manner, and a delayed diagnosis may lead to poor outcomes. The immunosuppressed state is a significant risk for hyperinfection in transplant recipients. Hence, a high clinical index of suspicion and early detection in these patients is crucial. This case prompted a review of our pre-transplant screening protocol for Strongyloides infection, mindful of the limitations of these tests. We now perform routine serological Strongyloides screening in all our potential transplant recipients, regardless of their clinical risk profile, although we recognise that serological testing would not have been useful in this case with persistently negative serological tests. Stratifying clinical risk and maintaining a high clinical index of suspicion in those at increased risk remains vital. More studies are needed to determine the optimal approach to both the screening for, and diagnosis of Strongyloides, particularly in those patients at highest risk, including transplant recipients and those with HIV.

## Data Availability

Not applicable.

## Abbreviations

ARVs: Antiretrovirals
3TC: Lamivudine
ddl: didanosine
LPV/r: Lopinavir/ritonavir
TB: Latent tuberculosis
CT: Computerised tomography
ELISA: Enzyme-linked immunosorbent assay
PCR: Polymerase chain reaction
